# Protocol: Reducing community violence: A systematic meta‐review of what works

**DOI:** 10.1002/cl2.1409

**Published:** 2024-05-19

**Authors:** David B. Wilson, Thomas Abt, Catherine Kimbrell, William Johnson

**Affiliations:** ^1^ Department of Criminology, Law and Society, Center for Evidence‐Based Crime Policy (CEBCP) George Mason University Fairfax Virginia USA; ^2^ Department of Criminology and Criminal Justice, The Center for the Study and Practice of Violence Reduction (VRC) University of Maryland College Park Maryland USA

**Keywords:** community violence, crime and violence, criminal and juvenile justice interventions and policies, overview of reviews

## Abstract

This is the protocol for a Campbell Collaboration systematic review. Our objective is to synthesize what is known about the effectiveness of strategies for reducing community violence, focusing on those strategies that have been subjected to a systematic review. We aim to answer the following questions in this review: what strategies to reduce community violence have been rigorously evaluated through systematic reviews; which have sufficient evidence of effectiveness, which seem promising, and which appear ineffective; and what implications for practice and policy can be drawn from this large body of research? We anticipate categorizing the results of our review similarly to the original review by Abt and Winship (2016). That is, categorizing reviews by people‐based approaches, place‐based approaches, and behavior‐based approaches. However, given that this is an updated review and we will be incorporating additional studies, we may find that an alternative or additional categorization is warranted and update our categorization accordingly. Implications for policy and practice as they relate to these categories will be discussed.

## 1 BACKGROUND

### 1.1 Description of the problem

The World Health Organization (WHO) has identified violence as a major public health challenge, impacting billions of lives each year through death, injury, and trauma (World Health Organization, 2023). The total global cost of violence in all its forms may be as high as 10% of the world's gross domestic product (Institute for Economics & Peace, 2022). The WHO defines violence as “the intentional use of physical force or power, threatened or actual, against oneself, another person, or against a group or community, that either results in or has a high likelihood of resulting in injury, death, psychological harm, maldevelopment or deprivation” (Krug et al., 2002, p. 5). For the purposes of the present review, we further refine this definition of violence and limit the scope of our review to what we term “community violence.” We conceptualize six dimensions of violent behavior (Abt & Winship, 2016). These six dimensions help define what is and what is not community violence.

For the first dimension, violence can vary in its lethality or capacity to cause serious physical injury (e.g., a shove vs. a fatal shooting). Second, violence can occur in different settings (e.g., in the privacy of one's home or on a public street). For the third dimension of violence, the number of individuals involved may be few, as with a dispute between neighbors, or many, as with conflicts among gangs. Fourth, violence may be spontaneous, such as in a bar brawl, or planned, such as an assassination. Fifth, violence may be expressive of an emotion, like anger, or instrumental, such as pursuing an illegal economic activity. For the sixth and final dimension, violence may be frequent (e.g., domestic violence) or infrequent (e.g., warfare) (Abt & Winship, 2016).

**Figure 1:**
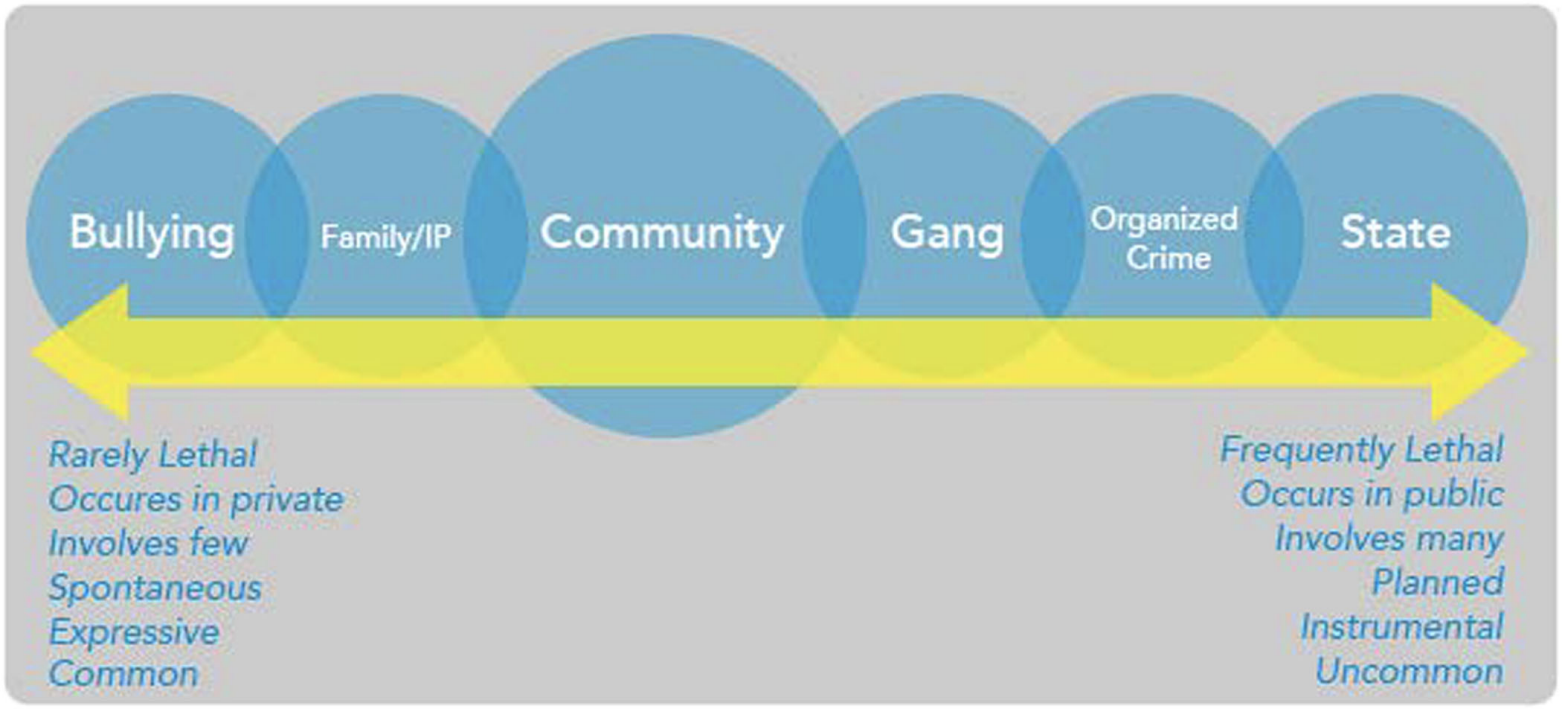
Continuum of violence. *Source*: Abt and Winship (2016). *What works in reducing community violence: a meta‐review and field study for the northern triangle*. Washington, DC: United States Agency for International Development.

Ultimately, we believe that violence can be viewed on a continuum based on the relationships between these six dimensions, ranging from bullying on one end of the spectrum to state‐level acts of violence on the other (see Figure 1) (Abt & Winship, 2016). In the middle of this continuum lies community violence. Community violence occurs primarily in public settings. It is interpersonal, taking place between individuals and small groups that may or may not know one another. It is generally unplanned and impulsive in nature, but its impact is nevertheless severe, often resulting in death or serious injury. Its perpetrators and victims are generally, but not exclusively, young men from disadvantaged backgrounds and communities (Centers for Disease Control and Prevention, 2022). It may result from disputes or conventional forms of street crime, such as robberies. Community violence implicates both the public health and public safety fields and can include a multi‐disciplinary, multi‐sector response (Abt & Winship, 2016). This is consistent with the Centers for Disease Control and Prevention's (CDC) definition of community violence as violence that “happens between unrelated individuals, who may or may not know each other, generally outside the home. Examples include assaults or fights among groups and shootings in public places” (Centers for Disease Control and Prevention, 2022, para. 1). Furthermore, the CDC notes that “youth and young adults (ages 10–34), particularly those in communities of color, are disproportionately impacted” (Centers for Disease Control and Prevention, 2022, para. 1). It is also consistent with the U.S. Department of Justice's definition, which describes community violence as “generally happening outside the home in public spaces” (Department of Justice, 2022, para. 1).

Drawing from these definitions, community violence, for the purpose of this review, is violence between individuals and groups that may or may not know one another in public (i.e., community settings). It results from interpersonal conflicts or from conventional forms of street crime, such as robbery, and its perpetrators and victims are often men from disadvantaged backgrounds and communities (Abt & Winship, 2016). What is excluded from this definition are bullying and intimate partner forms of violence on the left of the violence continuum. These forms of violence can be severe but generally occur in a private setting or an institution, such as a school. While bullying may occur in the community, we view it as sufficiently unique to be excluded from our definition of community violence. Similarly, we exclude state‐level violence, acts of terrorism, and mass shootings from our definition. No definition is perfect, but the boundary we draw is intended to capture routine forms of violence in the community.

Community violence is a particularly prevalent form of lethal violence (Abt, 2019). According to the Global Study of Homicide, one of the few publications to systematically assess these issues, non‐conflict violence causes roughly five times more deaths than those resulting from armed conflicts in most years. In 2021, 256,480 of 458,000 (56%) non‐conflict violent deaths were associated with some form of non‐domestic interpersonal violence or criminal violence (domestic violence was associated with 31% of non‐conflict violent deaths) (United Nations Office on Drugs and Crime, 2023).^1^ While the definitions do not match precisely, community violence was likely associated with the majority share of these 256,480 deaths, and with a similar number of deaths each year.

The costs of community violence are staggering. While the human costs of such tragedies are incalculable, the global economic cost of homicide and violent crimes in 2017 was approximately $3650 trillion, significantly greater than the global cost of collective violence, which includes deaths due to armed conflicts and terrorism, which is estimated at $1.02 trillion (Iqbal et al., 2021). In the United States, the American Hospital Association has estimated that community violence costs U.S. hospitals and health systems, a fraction of the total cost, approximately $2.7 billion in 2016 (Van Den Bos et al., 2017).

A relatively large body of rigorous evidence concerning strategies to reduce community violence is available, but it is fragmented, spread across hundreds of studies and dozens of reviews. This study will be a meta‐review, or review of reviews, to synthesize this evidence. We will examine systematic reviews of interventions that directly or indirectly address community violence. Implications for practice and policy will be examined and discussed. In responding to community violence, leaders must assess the suitability of a broad range of potential options. Our meta‐review will therefore provide a high‐level overview of the evidence across systematic reviews that collectively examine hundreds of programs, practices, and policies.

### 1.2 Description of the interventions

Our focus is not on any particular intervention. Rather, we are interested in any intervention, law, or social policy that might be adopted by a community or agency that is intended to reduce community violence. Because social programs often have multiple goals, the reduction of violence need not be the sole or even primary focus of the intervention. This focus is intentionally broad as our goal is to gather and consolidate the evidence based on the effectiveness of various activities that policymakers might select to reduce violence.

These interventions may focus their efforts on people, places, and/or specific behaviors. They may be implemented at the level of individuals, families, communities, organizations, or geographic areas such as neighborhoods, cities, states, or countries. Examples of potential interventions include: hot spots policing, neighborhood watch programs, cognitive‐behavioral programs, restorative justice programs, and juvenile curfews, to name but a few. Notice that some of these are intended to be rehabilitative for individuals already involved in crime, such as cognitive‐behavioral or restorative justice programs, while others are intended to have a general deterrent effect, such as hot spots policing and neighborhood watch programs. Others may be preventative, such as aftercare or mentoring programs for youth.

### 1.3 How the interventions might work

The current study will encompass a review of reviews of community violence interventions. Given the diversity of potential interventions eligible for inclusion in this study, ranging from place‐based to people‐based interventions, no single causal model could explain how or why these diverse interventions may work.

### 1.4 Why is it important to do this review?

The evidence base on interventions addressing community violence remains fragmented, as previously mentioned. As a result, this challenges the ability of practitioners and policymakers to confidently use evidence in decision‐making. In an effort to address this issue, Thomas Abt and Christopher Winship conducted a systematic meta‐review on “‘What Works’ in Reducing Community Violence: A Meta‐Review and Field Study for the Northern Triangle,” which was published in 2016. The co‐authors synthesized 43 reviews, including over 1400 studies from across the globe, to identify “what works” in reducing community violence. In addition, they supplemented their findings with fieldwork in El Salvador, Guatemala, Honduras, and the United States. However, since the release of the 2016 report, numerous systematic reviews on violence reduction have been published. This study, therefore, updates the meta‐review component of that report. Thus, we anticipate our meta‐review will have a larger evidence base from which to draw inferences.

Other relevant but not directly comparable reviews of anti‐violence strategies from around the world include Nation et al. (2003), Matjasko et al. (2012), Fazel et al. (2022), and Kovalenko et al. (2022). Three of these four prior reviews focused explicitly on violence prevention programs for youth or young people to varying degrees (Kovalenko et al., 2022; Matjasko et al., 2012; Nation et al., 2003). Nation et al. (2003) conducted a review‐of‐reviews, focusing specifically on identifying characteristics of effective youth prevention programs, while Matjasko et al. (2012) conducted a broader systematic meta‐review of evaluations of youth violence prevention programs. In a recent study, Kovalenko et al. (2022) conducted a systematic review‐of‐reviews on violence prevention among young people, focusing specifically on programs in an educational setting.

Fazel et al. (2022) was the only prior review that focused on violence prevention programs beyond just youth and young people. They conducted an umbrella review of meta‐analyses focusing on universal violence prevention interventions, or interventions aimed at the general population.

The current meta‐review is more focused than these prior studies in terms of the type of violence (i.e., community violence), but broader and more comprehensive in terms of the range of programs and practices eligible for inclusion, as it does not restrict the population of interest to youth or young people alone and does not restrict the intervention of interest solely to prevention programs.

## 2 OBJECTIVES

Our objective is to synthesize what is known about the effectiveness of strategies for reducing community violence, focusing on those strategies that have been subjected to a systematic review. We aim to answer the following questions in this review: what strategies to reduce community violence have been rigorously evaluated through systematic reviews; which have sufficient evidence of effectiveness, which seem promising, and which appear ineffective; and what implications for practice and policy can be drawn from this large body of research?

We anticipate categorizing the results of our review similarly to the original review by Abt and Winship (2016): categorizing reviews by people‐based approaches, place‐based approaches, and behavior‐based approaches. However, given that this is an updated review and we will be incorporating additional studies, we may find that an alternative or additional categorization is warranted, so we will update our categorization accordingly. Implications for policy and practice as they relate to these categories will be discussed.

## 3 METHODS

### 3.1 Criteria for considering studies for this review

#### 3.1.1 Types of studies

Eligible manuscripts must report on the results of a systematic review. The essential features are: (a) a systematic search for published and/or unpublished studies, (b) explicit eligibility criteria, (c) a coding protocol for extracting study results, and (d) an assessment of the quality of the evidence, such as assessing for risk of bias. The presence of each element is required, but the quality of each of these is not determinative of inclusion. Eligible manuscripts may report on the results of a meta‐analysis, but that is also optional for inclusion.

Eligible manuscripts must include studies assessing an eligible intervention's quantitative impact or effectiveness (see item 2 below). These might consist of experimental designs involving random assignment of experimental units to conditions, such as a randomized controlled trial, quasi‐experimental designs with or without comparison conditions, or observational studies that are analyzed in a way that provides a treatment effect estimate, such as an instrumental variable model. The critical feature is that the designs included in the review were (a) quantitative and (b) produced estimates of effectiveness. An eligible manuscript may also have other designs that do not meet these criteria.

#### 3.1.2 Types of interventions

Eligible manuscripts must review the effectiveness of an intervention (i.e., treatment, intervention, program, practice, social policy) intended to reduce crime and/or violence. For the purposes of this review, any meta‐analysis or systematic review that includes an eligible outcome and examines the effects of an intervention on an eligible intervention is eligible. An intervention is broadly defined as an action, program, or change to the status quo. That is, it is something manipulable by agencies or members of the public. Eligible interventions may be implemented at the level of individuals, families, communities, organizations, or geographic areas such as neighborhoods, cities, states, or countries. We will exclude interventions focusing on intimate partner and domestic violence.

#### 3.1.3 Types of participants

Interventions may involve other individuals, families, communities, organizations, or geographic areas. Essentially, any individual is eligible so long as the intervention is eligible. No restrictions will be placed on sex, race/ethnicity, and age.

#### 3.1.4 Types of outcome measures

Our primary outcome of interest is community violence (as defined in Section 1). Secondary outcomes include measures of general crime that are inclusive of violence. More specifically, eligible manuscripts will report the effectiveness of an eligible intervention on any of the following outcomes: (a) official measures of general crime (i.e., any crime), (b) official measures of violent crime, (c) victim reports of crime and/or violence, such as through surveys, (e) offender self‐report of crime and/or violence, and (f) third‐person reports of an offender's engagement in crime and/or violence, such as a parent. Systematic reviews are ineligible if crime measures exclude person (violent) crimes or include only measures of intimate partner or domestic violence. Any source (e.g., official, self‐report, other‐report) or indicator (e.g., arrests, charges, convictions, etc.) is eligible. Any review that includes the primary or secondary outcomes of community violence, violence generally, or crime generally is eligible.

#### 3.1.5 Other inclusion criteria

We will consider systematic reviews with a manuscript date of 1990 through 2023. The systematic reviews may have any temporal range for the included primary studies. Systematic reviews published prior to 1990 are likely to have study samples or topics covered by newer reviews.

Eligible manuscripts must be written in English, Spanish, Portuguese, or German. Spanish language studies are of particular interest due to the impact of community violence in some Spanish‐speaking countries, particularly the Northern Triangle (El Salvador, Guatemala, and Honduras). We will also include Portuguese manuscripts as Brazilian scholars have been working in the area of violence reduction and our team has the capacity to include this language. In addition, a member of the research team speaks German. Therefore, German studies will also be included to expand the potential scope of eligible studies. We will not restrict the publication status of manuscripts. Thus, both published and unpublished (gray literature) reviews will be eligible.

We will place no restrictions on the geographic scope of eligible systematic reviews.

### 3.2 Search methods for identification of studies

Our search strategy is intended to identify all eligible systematic reviews meeting the above eligibility criteria. It will consist of keyword searches of numerous databases and a hand search of selected journals. The keywords and Boolean logic for the database searches are as follows:

Set 1: meta‐analy* OR “meta analy*” OR “systematic review” OR “meta review” OR “analytical review” OR “quantitative review” OR “realist review” OR metaanaly*

Set 2: crime* OR “public disorder” OR victim* OR violen* OR delinquen* OR offen* OR police* OR “law enforcement” OR arrest* OR convict* OR gang* OR (community AND disorder) OR (youth AND crime*) OR (youth AND adjudicat*) OR (public AND disorder)

Set 3: interven* OR policy OR policies OR program* OR rehabilitat* OR treatment*

Set 4: Set 1 AND Set 2 AND Set 3 AND published 1990 forward

#### 3.2.1 Electronic searches

The list of databases that will be searched is as follows:

1. APA PsycExtra (via EBSCO)
2. APA PsycInfo (via EBSCO)
3. Australian Institute of Criminology
4. Book Citation Index – Social Sciences and Humanities (Web of Science)
5. Campbell Systematic Reviews Journal (http://campbellcollaboration.org)
6. Cochrane Database of Systematic Reviews (https://www.cochranelibrary.com)
7. Conference Proceedings Citation Index – Social Sciences and Humanities (Web of Science)
8. Criminal Justice Abstracts (via EBSCO)
9. Criminal Justice Database (via ProQuest)
10. Dissertations & Theses Full Text (via ProQuest)
11. EconLit (via EBSCO)
12. Education Resources Information Clearinghouse (ERIC) (via ProQuest)
13. JSTOR
14. National Criminal Justice Reference Service (NCJRS) (via EBSCO)
15. Psychology Database (via ProQuest)
16. PubMed
17. Social Science Citation Index (via Web of Science)
18. Social Science Database (via ProQuest)
19. Social Science Research Network (SSRN) eLibrary (https://www.ssrn.com/index.cfm/en/)
20. Sociological Abstracts (via ProQuest)
21. Sociology Database (via ProQuest)

Given the unique features of each database, we will restrict our search to titles, abstracts, keywords, and subject fields as appropriate to each database. An example of our search strategy as implemented in ProQuest can be found in Supporting Information: Appendix 1.

The list of Spanish‐language databases that will be searched is as follows:

1. Dialnet

The list of Portuguese‐language databases that will be searched is as follows:

1. Biblioteca Digital Brasileira de Teses e Dissertações (BDTD)
2. Catálogo de Teses e Dissertacoes da CAPES
3. Periódicos de CAPES
4. Biblioteca Virtual sobre Violencia e Sude da Biblioteca Regional de Medicina (BIREME)

The list of both Spanish and Portuguese‐language databases that will be searched is as follows:

1. Scientific Electronic Library Online (Biblioteca Científica Electrónica en Línea) (SciELO)
2. Red de revistas científicas de Acceso Abierto no comercial propiedad de la academia (Redalyc)
3. Sistema Regional de Información en línea para Revistas Científicas de América Latina, el Caribe, España y Portugal (Latindex)
4. Directory of Open Access Journals (DOAJ)
5. Academic journal index for the HSS (Humanities and Social Sciences) society in Europe (ErihPlus)
6. Scientific and academic journals published in Latin America and the Caribbean (Biblat)

#### 3.2.2 Searching other resources

In addition to the electronic database searches, we will also conduct separate hand searches of relevant journals. The list of journals that will be hand‐searched is as follows:

1. *Criminology*
2. *Criminology & Public Policy*
3. *Journal of Research in Crime and Delinquency*
4. *Journal of Criminal Justice*
5. *Police Quarterly*
6. *Policing*
7. *Police Practice and Research*
8. *Journal of Quantitative Criminology*
9. *Crime & Delinquency*
10. *Policing and Society*
11. *Journal of Interpersonal Violence*
12. *Trauma, Violence, and Abuse*

The prior meta‐review, which the present study is based on, concluded its search strategy in 2015 (Abt & Winship, 2016). The search period for the present study will, therefore, be from 2015 to the spring of 2023, when the search strategy commences for the updated study. As such, the search will begin with the last issue of each journal from 2015, as the previous hand search ended in the early fall of 2015. We will continue the search with each journal issue from 2016 through 2022, and the journal issues from 2023 up to when the search will be conducted in the spring of 2023. The results of the hand search will be logged in an Excel spreadsheet, tracking how many articles were screened and how many were retained as potentially eligible.

To minimize the potential for selection bias, we will search for gray literature studies (e.g., dissertations and theses, technical reports, non‐governmental reports, etc.). Toward this end, we will search the following databases and resources:

- Criminal Justice Abstracts (via EBSCO)
- Dissertations & Theses Full Text (via ProQuest)
- Evidence for Policy and Practice Information & Coordinating Center (EPPI‐Center) (https://eppi.ioe.ac.uk/cms/)
- National Criminal Justice Reference Service (NCJRS, via EBSCO)
- Prevention Institute (https://preventioninstitute.org)
- RAND Corporation (https://www.rand.org/pubs.html)
- Scottish Institute for Policing Research (SIPR) (https://www.sipr.ac.uk/researchreports/)
- Urban Institute (https://www.urban.org/research)
- What Works Crime Reduction Clearinghouse (https://whatworks.csgjusticecenter.org/)
- Youth Endowment Fund (YEF) toolkit (https://youthendowmentfund.org.uk/)

NCJRS and Criminal Justice Abstracts, in particular, both cover a wide range of gray literature and resources (e.g., Crimesolutions.gov, Australian Institute of Criminology, Youth Blueprints, National Institute of Justice [NIJ], Office of Juvenile Justice and Delinquency Prevention [OJJDP], etc.).

Finally, once we have retrieved all eligible studies, we will email the contact authors from the eligible studies. We will provide the authors with the list of studies we have compiled thus far and ask if they are aware of any additional studies that we did not include in our list. This will be the final step in our search process.

### 3.3 Data collection and analysis

#### 3.3.1 Selection of studies

Records identified in this search will be uploaded into Rayyan^2^ for initial screening. A copy of the text file version of the downloaded results will be retained. Rayyan allows for the importing of journal titles and abstracts for evaluation. As a first step, Rayyan offers the ability to run a search for duplicate articles. This process will be used, and the list of potential duplicates will be evaluated with duplicate entries deleted. Two project members will then screen the remaining list with a decision to include, exclude, or mark as maybe if the team member is unsure if the article should be included. Team members will not be able to see the screening decisions made by the other team members, insuring the independence of decisions. Conflicts in screening decisions will first be discussed between team members at set meetings, and if a disagreement remains, a third team member will evaluate. Decisions at this stage will err on the side of inclusion, and the decision will be made using the title and abstract only, not the full text.

We will obtain, if possible, the full text for all references marked as “include” or “maybe” in the above screening process. Two independent coders will evaluate each against the full eligibility criteria detailed in Section 3.1. This will be done using REDCap (see Section 3.3.4 below for more on this program) and any disagreements resolved by Wilson, Abt, or Kimbrell.

#### 3.3.2 Data extraction and management

We have developed a coding protocol to extract selected characteristics of eligible studies. We will code for review‐level characteristics, as well as the results, of each included review. Review level characteristics will include information on the publication itself (e.g., type of publication, year published, countries of authors, etc.), the intervention evaluated (e.g., the target of the intervention, age of people affected by the intervention, intervention characteristics, etc.), as well as methodology characteristics (e.g., number of studies included, research design employed, etc.). We will also code the results of each review, including mean effect sizes, if reported, for primary and secondary outcomes of interest, as well as other characteristics of the results (e.g., type of effect size, meta‐analysis method, narrative conclusion regarding the result, etc.). Please see Supporting Information (Appendix 2) for our full coding protocol.

We will follow a double coding process, whereby two coders will code each manuscript separately. Coding will take place in REDCap.^3^ REDCap is a free, secure, and online database, which makes it ideal for multiple coders to access the database and code at the same time. It also has a double coding function, which will help with easily identifying any coding differences. See Supporting Information: Appendix 3 for an example of our coding protocol in REDCap. Once coding differences are identified, the coders will conduct a reconciliation process, whereby differences will be discussed, and the coders will return to the original manuscript in question to reconcile any differences. If the coders are unable to reconcile their differences or need assistance, questions will be brought to the larger research team for a consensus discussion. Finally, we will calculate inter‐rater reliability for both the coding and screening stages.

#### 3.3.3 Assessment of risk of bias in included studies

We will assess the quality of the eligible systematic reviews using the AMSTAR‐2 (A MeaSurement Tool to Assess systematic Reviews) instrument.^4^ AMSTAR‐2 is a comprehensive critical appraisal instrument used for assessing the methodological quality of systematic reviews (Shea et al., 2017). It can be used to appraise systematic reviews of both randomized and non‐randomized studies with or without a meta‐analysis component. The AMSTAR‐2 checklist includes 16 items and will be completed for each manuscript. It will also be double‐coded in the REDCap database. The AMSTAR‐2 checklist is also provided in our Coding Protocol (see Supporting Information: Appendix 2 for details).

#### 3.3.4 Data synthesis

We will not perform a meta‐analysis across these mean effect sizes. There are two reasons for this. The first is that eligible meta‐analyses cover a vast range of interventions and are simply not meaningful at a policy or practice level whether they are, on average, effective. Thus, we want to maintain the granularity of the effectiveness of specific programs and interventions. Recall that each of these is based on a collection of studies. Second, interventions with multiple systematic reviews where it may make sense to produce a single overall estimate will include overlapping samples of primary studies, creating statistical dependencies among the related reviews.

Our approach to synthesizing the findings across systematic reviews will be to produce summary tables and forest plots (without an overall mean effect size) for conceptually related interventions and outcomes. An example might be a table and forest plot showing the findings for all geographically‐focused policing interventions. This may require harmonizing summary effect size statistics across meta‐analysis, such as transforming Hedges' *g* into an odds ratio, or visa‐versa. These tables and figures, along with the AMSTAR‐2 ratings, will provide the basis for conclusions drawn regarding the effectiveness of community violence reduction strategies.

A challenge we will face in our analysis is the issue of non‐independence across meta‐analyses. There are two main sources of this. The first and easiest address is multiple publications of the same systematic review. These may represent different analyses of the same meta‐analytic database or updates of prior reviews. In these instances, we will treat all publications that are distinct products from a common systematic review as a single entity, basing our coding on the most recent or up‐to‐date version. If needed, prior publications or manuscripts may be used to fill in missing information. The second is systematic reviews of a similar or overlapping topic. For example, we may identify multiple systematic reviews of hot spots policing conducted by independent research teams. These reviews will share many of the same studies but may also have studies that are unique to each review. Our review will include all such overlapping syntheses from independent research teams. We will not formally assess the degree of overlap, given that we are not performing any analysis that needs such an estimate. We will, however, assume a high degree of overlap for all such studies in drawing inferences about the effectiveness of an intervention with multiple systematic reviews. We will treat these as independent assessments of essentially the same (or highly similar) body of evidence. For example, if there are five reviews on the effectiveness of CBT for adult offenders, we will not treat these as five independent sources of evidence on the effectiveness of CBT. Rather, we will assume that these are replications of the systematic review process of overlapping literature.

## CONTRIBUTIONS OF AUTHORS

- *Content*: Thomas Abt, David B. Wilson, Catherine S. Kimbell, William Johnson
- *Systematic review methods*: David B. Wilson and Catherine S. Kimbrell
- *Statistical analysis*: David B. Wilson
- *Information retrieval*: Thomas Abt, David B. Wilson, Catherine S. Kimbell, and William Johnson
- *Screening and coding*: William Johnson, David B. Wilson, Catherine S. Kimbrell, and Richard Hahn
- *Drafting of reports*: All authors
- *Editing of reports*: All authors

## DECLARATIONS OF INTEREST

None of the authors have financial conflicts of interest. Thomas Abt was a co‐author of the original meta‐review on which the current study is based (Abt & Winship, 2016). Drs. Wilson and Kimbrell were authors of several meta‐analyses that may be eligible for the present study. Nevertheless, we do not anticipate this interfering with the meta‐review and selection process of eligible meta‐analyses. In addition, both Dr. Wilson and Thomas Abt have roles in the Campbell Collaboration's Crime & Justice Coordinating Group (CJCG). As such, they will not be privy to the internal editorial or approval processes for the review.

### 1 Preliminary timeframe

**Table:** 

Searches for published and unpublished studies	February–May 2023
Development of coding protocol and database	March–May 2023
Pilot testing and coding (including coding reconciliation)	May–April 2024
Analyses	April–May 2024
Preparation of final report	June 2024

### 2 Plans for updating this review

The review will be updated every 5 to 10 years. Thomas Abt will primarily lead these efforts.

## SOURCES OF SUPPORT

Arnold Ventures provided funding for this meta‐review.

### PEER REVIEW

The peer review history for this article is available at https://www.webofscience.com/api/gateway/wos/peer-review/10.1002/cl2.1409.

## Supporting information

Supporting information.

Supporting information.

Supporting information.

Supporting information.

## Footnotes

1 According to the study, homicides related to criminal activities are divided into homicides related to organized criminal groups and gangs, on the one hand, and homicides related to other criminal activities (e.g., robbery), on the other. Interpersonal homicides are divided into homicides perpetrated by intimate partners or other family members (domestic violence) and other interpersonal homicides perpetrated outside the family context (e.g., killings perpetrated by acquaintances or neighbors).

2
https://www.rayyan.ai/

3
https://projectredcap.org/

4
https://amstar.ca/index.php

## References

[cl21409-bib-0001] Abt, T. (2019). *Bleeding out: The devastating consequences of urban violence—and a bold new plan for peace in the streets*. Hachette UK.

[cl21409-bib-0002] Abt, T. , & Winship, C. (2016). What works in reducing community violence: A meta‐review and field study for the northern triangle. United States Agency for International Development.

[cl21409-bib-0003] Abt, T. P. (2017). Towards a framework for preventing community violence among youth. Psychology, Health & Medicine, 22(suppl 1), 266–285. 10.1080/13548506.2016.1257815 27973915

[cl21409-bib-0004] Centers for Disease Control and Prevention . (2022). *Community violence prevention*. Retrieved June 14, 2023 from https://www.cdc.gov/violenceprevention/communityviolence/index.html

[cl21409-bib-0005] Department of Justice . (2022). *Community Based Violence Intervention and Preventative Initiative (CVIPI)*. Retrieved June 21, 2023, from https://bja.ojp.gov/program/community-violence-intervention/overview

[cl21409-bib-0006] Fazel, S. , Burghart, M. , Wolf, A. , Whiting, D. , & Yu, R. (2022). *Universal violence prevention interventions: Umbrella review of effectiveness meta‐analyses*. ResearchGate. 10.31234/osf.io/aywte PMC10913357 37650521

[cl21409-bib-0007] Institute for Economics & Peace . (2022). *Global Peace Index. Measuring peace in a complex world, Sydney, June 2022*. Retrieved June 14, 2023, from https://www.economicsandpeace.org/wp-content/uploads/2022/06/GPI-2022-web.pdf

[cl21409-bib-0008] Iqbal, M. , Bardwell, H. , & Hammond, D. (2021). Estimating the global economic cost of violence: Methodology improvement and estimate updates. Defence and Peace Economics, 32(4), 403–426.

[cl21409-bib-0009] Kovalenko, A. G. , Abraham, C. , Graham‐Rowe, E. , Levine, M. , & O'Dwyer, S. (2022). What works in violence prevention among young people?: A systematic review of reviews. Trauma, Violence & Abuse, 23(5), 1388–1404. 10.1177/1524838020939130 PMC9606003 32677554

[cl21409-bib-0010] Krug, E. G. , Mercy, J. A. , Dahlberg, L. L. , & Zwi, A. B. (2002). The world report on violence and health. The Lancet, 360(9339), 1083–1088. 10.1016/S0140-6736(02)11133-0 12384003

[cl21409-bib-0011] Matjasko, J. L. , Vivolo‐Kantor, A. M. , Massetti, G. M. , Holland, K. M. , Holt, M. K. , & Dela Cruz, J. (2012). A systematic meta‐review of evaluations of youth violence prevention programs: Common and divergent findings from 25 years of meta‐analyses and systematic reviews. Aggression and Violent Behavior, 17(6), 540–552. 10.1016/j.avb.2012.06.006 29503594 PMC5831140

[cl21409-bib-0012] Nation, M. , Crusto, C. , Wandersman, A. , Kumpfer, K. L. , Seybolt, D. , Morrissey‐Kane, E. , & Davino, K. (2003). What works in prevention: Principles of effective prevention programs. American Psychologist, 58(6–7), 449–456. 10.1037/0003-066X.58.6-7.449 12971191

[cl21409-bib-0013] Shea, B. J. , Reeves, B. C. , Wells, G. , Thuku, M. , Hamel, C. , Moran, J. , Moher, D. , Tugwell, P. , Welch, V. , Kristjansson, E. , & Henry, D. A. (2017). AMSTAR 2: A critical appraisal tool for systematic reviews that include randomised or non‐randomised studies of healthcare interventions, or both. BMJ, 358, j4008. 10.1136/bmj.j4008 28935701 PMC5833365

[cl21409-bib-0014] United Nations Office on Drugs and Crime . (2023). *Global study on homicide 2023*. Retrieved February 7, 2024, from https://www.unodc.org/unodc/en/data-and-analysis/global-study-on-homicide.html

[cl21409-bib-0015] Van Den Bos, J. , Creten, N. , Davenport, S. , Roberts, M. , & American Hospital Association . (2017). *Cost of community violence to hospitals and health systems: Report for the American Hospital Association*. Millman. Retrieved June 14, 2023, from https://www.ashe.org/system/files/2018-01/community-violence-report.pdf

[cl21409-bib-0016] World Health Organization . (2023). *World Health Organization Violence Prevention Unit: Approach, objectives, and activities, 2022–2026*. Retrieved June 14, 2023, from https://cdn.who.int/media/docs/default-source/documents/social-determinants-of-health/who_2022_plv_strategy_2022-2026_finalfile.pdf?sfvrsn=c819ff54_3&download=true

