# Supplementary material for: Protocol: Reducing community violence: A systematic meta‐review of what works
Source: Campbell Syst Rev. 2024 May 19;20(2):e1409. doi: 10.1002/cl2.1409 (PMC11103278; doi:10.1002/cl2.1409)
Supplement: Supplementary file 1 — Supporting information. [file CL2-20-e1409-s003.pdf]

## Appendix 1: Example of Search in ProQuest

Name: ProQuest
Searched for: ABSTRACT,TITLE,SUMMARY,SUBJECT(meta-analys?s OR "meta analysis" OR "systematic review" OR "meta review" OR "analytical review" OR "quantitative review" OR "realist review" OR metaanalysis)
AND
ABSTRACT,TITLE,SUMMARY,SUBJECT(crime* OR “public disorder” OR victim* OR violen* OR delinquen* OR offen* OR police* OR law enforcement OR arrest* OR conviction OR gang* OR (community AND disorder) OR (youth AND crime) OR (youth AND adjudicate) OR (public AND disorder))
AND
ABSTRACT,TITLE,SUMMARY,SUBJECT(interven* OR policy OR policies OR program* OR rehabilitat*)
Databases: Criminal Justice Database, Psychology Database, Social Science Database, Sociology Database, Dissertations and Theses Global, ERIC, Sociological Abstracts
