# Supplementary material for: Protocol: Reducing community violence: A systematic meta‐review of what works
Source: Campbell Syst Rev. 2024 May 19;20(2):e1409. doi: 10.1002/cl2.1409 (PMC11103278; doi:10.1002/cl2.1409)
Supplement: Supplementary file 2 — Supporting information. [file CL2-20-e1409-s002.pdf]

Appendix 3: Example of RedCap Coding

Editing existing Reference ID **1**.

Save & Exit Form

Save & ...

– Cancel –

**Reference ID**	1
**Coder**	
**Study ID**	
**Reference IDs**	
**Publication type**	
**Year of publication**	Code actual year
**Countries of authors**	☐ US<br>☐ UK (England, Wales, Scottland)<br>☐ Netherlands<br>☐ Canada<br>☐ Spain<br>☐ Germany<br>☐ Israel<br>☐ Australia<br>☐ Other<br>Code the country of all authors. Select all that apply. If one author has multiple affiliations, also select both.
**Publication language**	Choose language publication was written in
**Author last names**	Format as: Name, Name, & Name. Include first name initial if same last names.
**Label for the intervention**	Short description of the intervention for the review
**Target of intervention: Primarily people or placed based**	Does the intervention target a place or people? One indicator of
