# Supplementary material for: Protocol: Reducing community violence: A systematic meta‐review of what works
Source: Campbell Syst Rev. 2024 May 19;20(2):e1409. doi: 10.1002/cl2.1409 (PMC11103278; doi:10.1002/cl2.1409)
Supplement: Supplementary file 3 — Supporting information. [file CL2-20-e1409-s001.pdf]

Appendix 2: Coding Protocol

Data Dictionary Codebook

Violence Prevention Systematic Review 2023

Instruments

	#	Variable / Field Name	Field Label *Field Note*	Field Attributes (Field Type, Validation, Choices, Calculations, etc.)
**Instrument: Study Level Characteristics (study_level_characteristics)**				
	1	[study_id]	Study ID<br>*Unique Study ID*	text
	2	[sl_coder]	Coder	dropdown<br>1 William Johnson<br>2 Catherine Kimbrell<br>3 David Wilson<br>4 Richard Hahn<br>5 Thomas Abt
	3	[reference_id]	Reference ID<br>*List all relevant reference IDs, including the reference for the study being coded (no commas)*	text
	4	[publication_type]	Publication type	dropdown<br>1 Journal<br>2 Campbell Review<br>3 Government/technical report<br>4 NGO or non-governmental technical report<br>5 Dissertation/thesis<br>6 Conference paper<br>7 Other published<br>8 Other unpublished
	5	[pub_year]	Year of publication<br>*Code year of publication*	text
	6	[author_countries]	Countries of authors<br>*Code the country of all authors. If one author has multiple affiliations, also select both. Do primary author and move on IF all countries not listed for other authors.*	checkbox<br>1 author_countries___1 US<br>2 author_countries___2 UK (England, Wales, Scottland)<br>3 author_countries___3 Netherlands<br>4 author_countries___4 Canada

				5 author_countries___5 Spain<br>6 author_countries___6 Germany<br>7 author_countries___7 Israel<br>8 author_countries___8 Australia<br>9 author_countries___9 Other
	7	[author_country_other]<br><br>Show the field ONLY if:<br>[author_countries(9)] = '1'	County author other (please write out)	text
	8	[pub_language]	Publication language<br>*Choose language publication was written in*	dropdown<br>1 English<br>2 Spanish<br>3 German<br>4 Portugese<br>5 Other
	9	[pub_language_other]<br><br>Show the field ONLY if:<br>[pub_language] = '5'	Publication language other (please write out)	text
	10	[author_names]	Author last names<br>*Format as: Name, Name, & Name. Include first name initial if same last names.*	text
	11	[intervention]	Label for the intervention<br>*Short description of the intervention for the review*	text
	12	[intervention_target]	Target of intervention: Primarily people or placed based<br>*Does the intervention target a place or people? One indicator of this is whether data are collected on a sample of individuals or on a sample of geographic areas.*	dropdown<br>1 People<br>2 Places<br>3 Other<br>4 Law/Policy/Legislation
	13	[intervention_target_other]<br><br>Show the field ONLY if:<br>[intervention_target] = '3'	Target of intervention other (please write out)	text
	14	[place_size]<br><br>Show the field ONLY if:	If place-based, size of area (for focus of intervention).<br>*Micro places are small geographic areas such as a street intersection, hot spots, etc. Meso places are*	dropdown<br>1 Micro<br>2 Meso

<table>
<tr><td></td><td></td><td>[intervention_target] = '2'</td><td>middle-sized geographic areas, such as neighborhoods; police district; subdivided section of a city; community. Macro places are large geographic areas such as a city, state, county.</td><td><table><tr><td>3</td><td>Macro</td></tr></table></td></tr>
<tr><td></td><td>15</td><td>[people_unit]

Show the field ONLY if:
[intervention_target] = '1'</td><td>If people based, unit of intervention focus:
(check all that apply)</td><td>checkbox<table><tr><td>1</td><td>people_unit___1</td><td>Individuals</td></tr><tr><td>2</td><td>people_unit___2</td><td>Families</td></tr><tr><td>3</td><td>people_unit___3</td><td>Individuals + Family</td></tr><tr><td>4</td><td>people_unit___4</td><td>Non-familial groups (e.g., gangs; peer groups, individuals who know each other)</td></tr><tr><td>5</td><td>people_unit___5</td><td>Sub-population (e.g. individuals who do not know each other, at risk individuals)</td></tr><tr><td>6</td><td>people_unit___6</td><td>General population</td></tr></table></td></tr>
<tr><td></td><td>16</td><td>[people_risk]

Show the field ONLY if:
[intervention_target] = '1'</td><td>If people-based, risk-level of individuals:
(check all that apply)</td><td>checkbox<table><tr><td>1</td><td>people_risk___1</td><td>Individuals known to have committed crime (tertiary; symptomatic/sick)</td></tr><tr><td>2</td><td>people_risk___2</td><td>Individuals at risk of committing crime (secondary prevention; elevated risk)</td></tr><tr><td>3</td><td>people_risk___3</td><td>General population (primary; at no elevated risk)</td></tr></table></td></tr>
<tr><td></td><td>17</td><td>[people_therapeutic]

Show the field ONLY if:
[intervention_target] = '1'</td><td>If people-based, is the program/intervention therapeutic?
The program focuses on improving individuals (or families, etc.) in some way that will result in a reduction in future criminal behavior.</td><td>dropdown<table><tr><td>1</td><td>Yes</td></tr><tr><td>2</td><td>No</td></tr><tr><td>3</td><td>Cannot tell</td></tr><tr><td>4</td><td>Some programs/interventions</td></tr></table></td></tr>
<tr><td></td><td>18</td><td>[people_value]</td><td>If people-based, does the program/intervention provide some</td><td>dropdown<table><tr><td>1</td><td>Yes</td></tr></table></td></tr>
</table>

		Show the field ONLY if: [intervention_target] = '1'	resource of value to participants?<br>*For example, job training, assistance with housing, etc. This does NOT include therapy.*	2 No<br>3 Cannot tell<br>4 Some programs/interventions
	19	[people_sanction]<br>Show the field ONLY if: [intervention_target] = '1'	If people-based, does the program/intervention include sanctions?<br>*Sanctions are punitive in some way and are designed to suppress unwanted behaviors, such as violence or other criminal behavior.*	dropdown<br>1 Yes<br>2 No<br>3 Cannot tell<br>4 Some programs/interventions
	20	[int_single_behavior]	If people- or place-based, does the intervention focus on a single behavior, such as gun carrying or drug dealing?	dropdown<br>1 Yes<br>2 No<br>3 Cannot tell
	21	[int_behavior_describe]<br>Show the field ONLY if: [int_single_behavior] = '1'	Describe behavior	notes
	22	[age]<br>Show the field ONLY if: [intervention_target] = '1' or [intervention_target] = '3'	Age of people affected by intervention (select all that apply).<br>*The age thresholds are also not rigid. Thus, if a program is for children up through age 11, select "kids/children (below 10)". The idea is to code this in a manner that best describes the participants of the program(s) examined by the systematic review.*	checkbox<br>1 age___1 Kids/children (below ~10)<br>2 age___2 Adolescents (~10-17)<br>3 age___3 Young adults (generally ~18-25)<br>4 age___4 All adults (18+)<br>5 age___5 All (general population). Note: If all age groups apply, select this instead of selecting all of the specific age groups.
	23	[int_population]<br>Show the field ONLY if: [intervention_target] = '1' or [intervention_target] = '3'	Targeted population of intervention	checkbox<br>1 int_population___1 Female only<br>2 int_population___2 Male only<br>3 int_population___3 Homeless<br>4 int_population___4 Mentally ill<br>5 int_population___5 Substance addicted<br>6 int_population___6 Gang affiliated

<table>
<tr><td></td><td></td><td></td><td></td><td><table>
<tr><td>7</td><td>int_population___7</td><td>Specific race only</td></tr>
<tr><td>8</td><td>int_population___8</td><td>Non-specific (i.e., all juvenile delinquents)</td></tr>
<tr><td>9</td><td>int_population___9</td><td>Other</td></tr>
</table></td></tr>
<tr><td></td><td>24</td><td>[int_population_race_other]

Show the field ONLY if:
[int_population(7)] = '1'</td><td>Specific race only - please specify</td><td>text</td></tr>
<tr><td></td><td>25</td><td>[int_population_other]

Show the field ONLY if:
[int_population(9)] = '1'</td><td>Other - please specify.</td><td>text</td></tr>
<tr><td></td><td>26</td><td>[int_setting]</td><td>Setting of intervention (Select all that apply)
Where is the intervention occuring?</td><td>checkbox<table>
<tr><td>1</td><td>int_setting___1</td><td>Carceral</td></tr>
<tr><td>2</td><td>int_setting___2</td><td>School</td></tr>
<tr><td>3</td><td>int_setting___3</td><td>Hospital</td></tr>
<tr><td>4</td><td>int_setting___4</td><td>Community (inside building)</td></tr>
<tr><td>5</td><td>int_setting___5</td><td>Community (outside)</td></tr>
<tr><td>6</td><td>int_setting___6</td><td>Other</td></tr>
</table></td></tr>
<tr><td></td><td>27</td><td>[int_setting_other]

Show the field ONLY if:
[int_setting(6)] = '1'</td><td>Setting other (please specify)</td><td>text</td></tr>
<tr><td></td><td>28</td><td>[int_provider]</td><td>Provider of intervention (Select all that apply)
Who is actually doing the intervention?</td><td>checkbox<table>
<tr><td>1</td><td>int_provider___1</td><td>Criminal justice org</td></tr>
<tr><td>2</td><td>int_provider___2</td><td>Other governmental org</td></tr>
<tr><td>3</td><td>int_provider___3</td><td>Business org</td></tr>
<tr><td>4</td><td>int_provider___4</td><td>Non-profit org</td></tr>
<tr><td>5</td><td>int_provider___5</td><td>Other</td></tr>
</table></td></tr>
</table>

	29	[int_provider_other]<br><br>Show the field ONLY if:<br>[int_provider(5)] = '1'	Provider other (please specify)	text
	30	[int_category]	Intervention category (Select all that apply)	checkbox<br>1 int_category___1 Aftercare programs<br>2 int_category___2 Boot camp<br>3 int_category___3 Cognitive behavioral therapy<br>4 int_category___4 Community-oriented policing<br>5 int_category___5 Comprehensive gang reduction<br>6 int_category___6 Comprehensive gun violence reduction<br>7 int_category___7 Crime Prevention Through Environmental Design (CPTED)<br>8 int_category___8 Disorder (broken windows) policing<br>9 int_category___9 Drug courts and treatment<br>10 int_category___10 Drug enforcement<br>11 int_category___11 Electronic monitoring<br>12 int_category___12 Family-based programs<br>13 int_category___13 Focused deterrence<br>14 int_category___14 Gang behavior regulation<br>15 int_category___15 Gang prevention<br>16 int_category___16 Gun buybacks

				17, int_category___17, Gun enforcement<br>18, int_category___18, Gun legislation<br>19, int_category___19, Hot spots policing<br>20, int_category___20, Juvenile curfews<br>21, int_category___21, Mentoring<br>22, int_category___22, Neighborhood watch<br>23, int_category___23, Poverty deconcentration<br>24, int_category___24, Problem-oriented policing<br>25, int_category___25, Procedural justice<br>26, int_category___26, Restorative justice<br>27, int_category___27, Risk-Need Responsivity (RNR) framework<br>28, int_category___28, Scared straight<br>29, int_category___29, School-based programs<br>30, int_category___30, Urban renewal<br>31, int_category___31, Vocational training<br>32, int_category___32, Other
	31	[int_category_other]<br>Show the field ONLY if:<br>[int_category(32)] = '1'	Intervention category other (please write out)	text
	32	[study_num]	Number of studies in review<br>*Code the total number of independent studies included in the review*	text
	33	[evaluations]	Number of unique evaluations	text
	34	[studies_usa]	Number of studies conducted in the United States	text

<table>
<tr><td></td><td>35</td><td>[study_design]</td><td>Research designs eligible for inclusion (check all that apply).
Check all that apply based on eligibility criteria</td><td>checkbox<table><tr><td>1</td><td>study_design___1</td><td>RCTs</td></tr><tr><td>2</td><td>study_design___2</td><td>All quasi-experiments with a comparison group</td></tr><tr><td>3</td><td>study_design___3</td><td>Selected quasi-experiments with comparison group (i.e. DiD, matching, baseline controls, etc.)</td></tr><tr><td>4</td><td>study_design___4</td><td>Non-RCT, one-group pre-test/posttest</td></tr><tr><td>5</td><td>study_design___5</td><td>Interrupted time series</td></tr><tr><td>6</td><td>study_design___6</td><td>Regression discontinuity</td></tr><tr><td>7</td><td>study_design___7</td><td>Other</td></tr></table></td></tr>
<tr><td></td><td>36</td><td>[study_design_other]
Show the field ONLY if:
[study_design(7)] = '1'</td><td>Research design other (please write out)</td><td>text</td></tr>
<tr><td></td><td>37</td><td>[num_rct]</td><td>Number of RCT designs included in review</td><td>text</td></tr>
<tr><td></td><td>38</td><td>[cost_analysis]</td><td>Cost benefit analysis included</td><td>yesno<table><tr><td>1</td><td>Yes</td></tr><tr><td>0</td><td>No</td></tr></table></td></tr>
<tr><td></td><td>39</td><td>[vote_counting]</td><td>Vote counting?</td><td>yesno<table><tr><td>1</td><td>Yes</td></tr><tr><td>0</td><td>No</td></tr></table></td></tr>
<tr><td></td><td>40</td><td>[study_narrative]</td><td>Narrative conclusion regarding this study. What overall conclusion did the authors draw from this study? Ideally please use a direct quote and include page number.</td><td>notes</td></tr>
<tr><td></td><td>41</td><td>[study_notes]</td><td>Notes/comments</td><td>notes</td></tr>
<tr><td></td><td>42</td><td>[groupings]</td><td>Grouping</td><td>text</td></tr>
</table>

	43	[study_level_characteristics_complete]	Section Header: *Form Status*<br>Complete?	dropdown<br>0 Incomplete<br>1 Unverified<br>2 Complete
**Instrument: Amstar2 (amstar2)**				
	44	[amstar_coder]	Coder	dropdown<br>1 William Johnson<br>2 Catherine Kimbrell<br>3 David Wilson<br>4 Sarah Silberman<br>5 Elliott Masouredis<br>6 Thomas Abt<br>7 Vivian
	45	[amstar_1]	1. Did the research questions and inclusion criteria for the review include the components of PICO?	dropdown<br>1 Yes<br>2 No
	46	[amstar_4]	Section Header: *1. Our criteria: For Yes: Discussed eligibility criteria Criteria included information related to Population Intervention Comparator group (could be "treatment as usual") Outcome(s) Original criteria: For Yes: Population Intervention Comparator group Outcome*<br>4. Did the review authors use a comprehensive literature search strategy?	dropdown<br>1 Yes<br>2 No<br>Custom alignment: RH
	47	[amstar_6]	Section Header: *4. Our modified criteria: Yes (all of the following): Searched at least 2 databases (relevant to research question) Provided key word and/or search strategy Search included attempt to find grey literature studies Justified any geographic, linguistic, timeframe restrictions unless such a justification seems unnecessary (only studies conducted in the US when examining an intervention that is specific to the US context) Original criteria: For Partial Yes, (all the following): Searched at least 2 databases (relevant to research question) Provided key word and/or search strategy Justified publication restrictions (e.g. language) For Yes, should also have all the following: Included/consulted content experts in the field where relevant, searched for grey literature Conducted search within 24 months of completion of the review Searched the reference lists/bibliographies of included studies Searched trial/study registries*<br>6. Did the review authors perform data extraction in duplicate?	dropdown<br>1 Yes<br>2 No
	48	[amstar_8]	Section Header: *6. For Yes, either ONE of the following: At least two reviewers achieved consensus on which data to extract from included studies OR*	dropdown<br>1 Yes

			two reviewers extracted data from a sample of eligible studies and achieved good agreement (at least 80 percent), with the remainder extracted by one reviewer.<br>8. Did the review authors describe the included studies in adequate detail?	2 No
	49	[amstar_9]	Section Header: *8. Our criteria: For Yes, provided one of the following: A table with descriptives for each study Summary descriptive statistics of study characteristics (i.e., for larger meta-analyses) Original criteria: For Partial Yes (all the following): Described populations Described interventions Described comparators Described outcomes Described research designs For Yes, should also have all the following Described population in detail Described intervention in detail (including doses where relevant) Described comparator in detail (including doses where relevant) Described study's setting Timeframe for follow-up*<br>9. Did the review authors use a satisfactory technique for assessing the risk of bias (RoB) in individual studies that were included in the review?	dropdown<br>1 Yes<br>2 No
	50	[amstar_11]	Section Header: *9. Our modified criteria: For Yes, must have assessed studies for RoB or design quality. This might include any of the following: Using an RoB tool Using the Maryland Scale of Scientific Methods Coded for various design features related to internal validity (e.g., random assignment, baseline equivalence, control for baseline differences, etc.) Original criteria: For Partial Yes, must have assessed RoB: From confounding, and From selection bias For Yes, must also have assessed RoB: Methods used to ascertain exposures and outcomes, and Selection of the reported result from among multiple measurements or analyses of a specified outcome*<br>11. If meta-analysis was performed did the review authors use appropriate methods for statistical combination of results?	dropdown<br>1 Yes<br>2 No<br>3 No meta-analysis conducted
	51	[amstar_12]	Section Header: *11. Our criteria: For Yes, did all of the following: Used an appropriate method of meta-analysis. These include inverse variance weighing, the Hunter and Schmidt method, or the Rosenthal method. Reported a measure of heterogeneity (Q, I^2, H^2, tau or tau^2). Used a random effects model unless a fixed effect model was well justified (note that the Hunter and Schmidt method is a random effects approach), or reported both a random effects and fixed effect model. Maintained statistical independence among effect sizes (one effect size per unique study sample) or model dependencies (i.e., robust variance estimation method or multi-level meta-analysis) Original criteria: For Yes: The authors justified combining the data in a meta-analysis AND they used an appropriate weighted technique to combine study results, adjusting for heterogeneity if present AND they statistically combined effect estimates from*	dropdown<br>1 Yes<br>2 No<br>3 No meta-analysis conducted

			NRSI that were adjusted for confounding, rather than combining raw data, or justified combining raw data when adjusted effect estimates were not available AND they reported separate summary estimates for RCTs and NRSI separately when both were included in the review<br>12. If meta-analysis was performed, did the review authors assess the potential impact of RoB in individual studies on the results of the meta-analysis or other evidence synthesis?	
	52	[amstar_13]	Section Header: *12. Our criteria: For Yes, must have done at least one of the following: Reported results separately for high/low RoB studies (i.e., RCTs vs quasi-experiments) Only included RCTs Performed a moderator analysis that assessed for method bias No RCTs and either too few studies for differential analysis OR no meaningful across study differences. Original criteria: For Yes: Included only low risk of bias RCTs Or, if the pooled estimate was based on RCTs and/or NRSI at variable RoB, the authors performed analyses to investigate possible impact of RoB on summary estimates of effect'*<br>13. Did the review authors account for RoB in individual studies when interpreting/ discussing the results of the review?	dropdown<br>1 Yes<br>2 No
	53	[amstar_14]	Section Header: *13. For Yes: Included only low risk of bias RCTs OR, if RCTs with moderate or high RoB, or NRSI were included the review provided a discussion of the likely impact of RoB on the results Note: The general issue here is whether they contextualize the findings in the context of the methodological quality of the studies.*<br>14. Did the review authors provide a satisfactory explanation for, and discussion of, any heterogeneity observed in the results of the review?	dropdown<br>1 Yes<br>2 No<br>3 No meta-analysis
	54	[amstar_15]	Section Header: *14. Our criteria: For Yes: There was no significant heterogeneity in the results OR if heterogeneity was present, conclusions acknowledged variability in effectiveness across studies (i.e., the conclusions appropriately reflected observed variability in results) OR results are inconclusive such that the authors do not arrive at a conclusion regarding the effectiveness of the intervention Original criteria: For Yes: There was no significant heterogeneity in the results OR if heterogeneity was present the authors performed an investigation of sources of any heterogeneity in the results and discussed the impact of this on the results of the review*<br>15. If they performed quantitative synthesis did the review authors carry out an adequate investigation of publication bias (small study bias) and discuss its likely impact on the results of the review?	dropdown<br>1 Yes<br>2 No<br>3 No meta-analysis conducted

	55	[yes]	15. Our criteria: For Yes, did one of the following: Performed graphical or statistical tests for publication bias and discussed the likelihood and magnitude of impact of publication bias Compared the results from published and gey literature studies and discussed the likelihood and magnitude of impact of publication bias If too few studies for any quantitative assessment of publication selection bias, acknowledge the possibility of such bias Original criteria: For Yes: Performed graphical or statistical tests for publication bias and discussed the likelihood and magnitude of impact of publication bias	descriptive
	56	[amstar_notes]	Notes/Comments	notes
	57	[amstar2_complete]	Section Header: *Form Status*<br>Complete?	dropdown<br>0 Incomplete<br>1 Unverified<br>2 Complete
**Instrument: Results (results)**				
	58	[es_coder]	Section Header: *Results info This is a repeating form. You can code as many results as are eligible for our review. Of interest is the overall result for general crime and violent crime. Do not code property crime and/or drug crimes if it is reported as a separate outcome. Only code non-official measures of crime if official measures are not available. Also, code results specific to distinct treatment or program types if reported. For example, a meta-analysis of CBT might report results separately from "reasoning and rehabilitation" and "anger replacement therapy". Findings might be one of three types: (1) a numeric meta-analytic result, such as a mean effect size and associated statistics; (2) a textual meta-analysis result, such as the findings of a moderator analysis that cannot be coded as a single mean effect size, such as from a meta-regression model; and (3) a textual systematic review finding. For type 2, only code those moderator analyses that reflect a main finding from the meta-analysis and would be useful to the adoption or implementation of a program of this type. Calculating SE! SE=ES/t SE=ES/z For youth/juvenile studies, only include checklist specific to juvenile violence OR conduct disorder if predominately deliquent behavior. For example, the CBCL has many sub-scales. We would not want general problem behaviors or externalizing behaviors. BUT the sub-scale that is specific to deliquency we would code.Put results in the order that they appear in text, ideally in the order found in tables.*<br>Coder	dropdown<br>1 William Johnson<br>2 Catherine Kimbrell<br>3 David Wilson<br>4 Richard Hahn<br>5 Thomas Abt

	59	[result_type]	Type of result:	radio<br>1 Meta-analysis result (numeric, text)<br>2 Meta-analysis result (text only)<br>3 Systematic-review result (text only)
	60	[es_label]	Label (short description) for this finding<br>*Write a label that would be suitable for a results table that will help differentiate this result from others coded for this study.*	text
	61	[es_construct_cat]	Outcome construct category	dropdown<br>1 Official measure - Criminal behavior (general)<br>2 Official measure - Criminal behavior (violence)<br>3 Official measure - Criminal behavior (other:)<br>4 Unofficial measure - violence/aggression<br>5 Unofficial measure - general problem behavior<br>6 Unofficial measure - other behaviors<br>7 Mixed (Official measure and unofficial (self-report))
	62	[es_construct_unoff_other]<br>Show the field ONLY if:<br>[es_construct_cat] = '6'	unofficial other (write out)	text
	63	[es_construct_cat_other]<br>Show the field ONLY if:<br>[es_construct_cat] ="3"	Label for other outcome construct category	text
	64	[es_unit]	Unit of measurement<br>*Micro places are small geographic areas such as a street intersection, hot spots, etc. Meso places are middle-sized geographic areas, such as neighborhoods; police district; subdivided section of a city; community. Macro places are large geographic areas such as a city, state, country.*	dropdown<br>1 Individuals<br>2 Place/micro<br>3 Place/meso<br>4 Place/macro<br>5 Other

	65	[es_unit_other]<br>Show the field ONLY if:<br>[es_unit] = '5'	Unit of measurement - other (please write in).	text
	66	[es_method]	Section Header: *Answer following questions for meta-analysis/effect size only.*<br>Meta-analysis method	radio<br>1 Fixed-effect<br>2 REML random effects<br>3 Dersimonian/Laird random effects<br>4 Random effects (estimation method unknown)<br>5 Farrington MVE<br>6 Not a meta-analysis (systematic review)<br>7 Other<br>8 Not described (unknown)
	67	[es_method_other]<br>Show the field ONLY if:<br>[es_method] = '7'	Meta-analysis method other (please write in)	text
	68	[es_sub_tx]	Is this effect a moderator analysis for a specific program or treatment type?Note: For moderator analyses, we do not want variation in the program (e.g. pre-adjudication, post-adjudication), but categorically different programs (e.g. adult drug court, juvenile drug court).	yesno<br>1 Yes<br>0 No
	69	[es_tx_sub_lbl]<br>Show the field ONLY if:<br>[es_sub_tx]="1"	If yes, label for this program or treatment type.	text
	70	[es_type]	Effect size type reported	radio<br>1 Cohen's d/Hedges' g (standardized mean difference)<br>2 Logged odds ratio<br>3 Unlogged odds ratio<br>4 Logged risk ratio<br>5 Unlogged risk ratio<br>6 Logged IRR/RIRR<br>7 Unlogged IRR/RIRR<br>8 Correlation (r)<br>9 Other

	71	[es_type_other]<br>Show the field ONLY if:<br>[es_type] = '9'	Effect size other (please write in)	text
	72	[es_favor]	Direction of effect	radio<br>1 Favors TX<br>2 Favors control<br>3 Cannot tell
	73	[es_mean_val]	Mean effect size value<br>*Actual mean effect size for this result*	text
	74	[es_se]	Standard error of mean<br>*Standard error for the mean*	text
	75	[es_lower_ci]	Lower CI of the mean<br>*Lower 95% CI for the mean*	text
	76	[es_upper_ci]	Upper CI of the mean<br>*Upper 95% CI for the mean*	text
	77	[es_n]	Number of effect sizes<br>*Number of effect sizes contributing to the mean*	text
	78	[es_studies]	Number of studies	text
	79	[es_q]	Heterogeneity Q<br>*This might be reported as a Chi^2*	text
	80	[es_tau2]	Tau-squared<br>*This might be reported as Tau (square root of Tau^2)*	text
	81	[es_i2]	I^2	text
	82	[z]	Z	text
	83	[es_narrative]	Section Header: *Result final questions (for both systematic reviews and meta-analyses)*<br>Narrative conclusion regarding this result. What conclusion did the authors draw regarding this outcome?<br>Preferably a direct quote w/ pg. number.	notes
	84	[pg_result]	Page result found	text
	85	[result_grouping]	Grouping	text
	86	[result_notes]	Notes/comments	notes
	87	[results_complete]	Section Header: *Form Status*<br>Complete?	dropdown<br>0 Incomplete<br>1 Unverified<br>2 Complete
