# Supplementary material for: Protocol: Reducing community violence: A systematic meta‐review of what works
Source: Campbell Syst Rev. 2024 May 19;20(2):e1409. doi: 10.1002/cl2.1409 (PMC11103278; doi:10.1002/cl2.1409)
Supplement: Supplementary file 4 — Supporting information. [file CL2-20-e1409-s004.docx]

**Consolidated reviews:** Reducing community violence: A meta-review of what works [Protocol]

**Campbell Collaboration Crime and Justice Coordinating Group**

**General points Feedback Reviewer Revised? Author comments ED Use Only**

| General Points | I’ve based my review on [the PRIOR statement](https://www.bmj.com/content/bmj/suppl/2022/08/09/bmj-2022-070849.DC1/gatm070849.ww3.pdf) for reporting overviews of reviews but as this relates to review reporting, some elements have been interpreted in the context of a protocol, or are not relevant. | ME |  | No revision is needed. |  |
| --- | --- | --- | --- | --- | --- |
| General Points | I commend the authors for undertaking a systematic review of reviews (i.e., a meta-review) on what works to prevent community violence. Meta-reviews are an important resource for the field and having one focused on summarizing the wide swath of systematic reviews and meta-analyses on community violence prevention is needed. Overall, I think that the protocol is methodologically strong. My comments are focused on tightening some key points in their protocol, advancing additional conceptual clarity, and encouraging the authors to integrate an equity assessment in their methods. There are also a few typos in the document so the authors should do a spell check before resubmitting the protocol for publication. My specific points are summarized below. | R1 |  | No revision is needed. |  |
| General Points | The language used is, in the main, very clear and easily understood.  The methods section is very clearly written. The authors indicate the types of studies and interventions that they will identify and synthesise as well as the types of outcome measure required for inclusion. The search strategy is comprehensive as are the methods for selecting studies, data extraction and synthesis. I feel this is a very timely and well thought out review which will build on the original 2016 Abt and Winship study.  This is a well-written, methodologically sound protocol which addresses an important problem. I look forward to reading the finished review. | R2 |  | No revision is needed. |  |
| Title | PRIOR suggests using “overview of reviews” in the title and the authors should consider this for consistency. | ME |  | We strongly prefer “meta-review” and are unaware of any Campbell policy on this issue. PRIOR is also specifically referring to health care interventions, and we are not in that context. |  |

**Background Feedback Reviewer Revised? Author comments ED Use Only**

| Description of the problem | I appreciate the authors’ thorough definition of community violence. Figure 1 is particularly helpful in mapping out the definition. I have a few additional comments and recommendations that could help to strengthen it further:   - - The following statement on page 2 needs a citation: “Community violence, particularly homicide, occurs primarily in public settings.” Is this statement referring to the prevalence of all homicides? Or, is this homicides that fall under the umbrella of community violence? If it is the former, then there is some variability in where homicides occur. For example, many occur in homes between people who know each other. The authors need to further clarify this and provide a citation.   - Citations are also needed to support the rest of their definition, including the unplanned nature of it, the severity of it, and the fact that young men of color are disproportionately involved in community violence. | R1 |  | This sentence refers to homicides that fit the definition of community violence. Note that we are excluding intimate partner violence (see below). We have deleted “particularly homicide” from this sentence, as it is not critical to our point.  We do not believe that we need to cite the definition in point 2, as it is ours. Others may develop a somewhat different definition.  We have removed “the unplanned nature of it,” as that is not critical to our definition. We do not claim that “young men of color are disproportionately involved” in community violence. We did say that “young men from disadvantaged backgrounds” are disproportionately involved. This was supported by a citation further down in the paragraph. We have now provided the citation directly after the claim. |  |
| --- | --- | --- | --- | --- | --- |
| Description of the problem | At times, the authors use the term “criminal violence” and I am not sure why. I encourage them to use consistent terminology throughout. If there is a different term that is being invoked, it needs to be clear why (e.g., is this a term that is used in the studies that they cite?). | R1 |  | We have fixed this where it made sense to do so.  We have left “criminal violence” in the discussion of the UNODC data, as that is about criminal violence, which is broader than community violence. |  |
| Description of the problem | On page 3, it would be helpful to noted how UNODC’s definition of “intentional homicide” is the same and different from the authors’ definition. This will help to put those numbers in better context. I also suggest using the per capita homicide rate to put the estimates from 1990 and 2017 in better context. | R1 |  | We have made edits to this section. |  |
| Description of the problem | The definition of community violence is clear, logical, and well justified using relevant citations. The scale of the challenge facing societies in relation to community violence is well described and provides a clear justification for this meta-review. | R2 |  | No revision is needed. |  |
| Description of the intervention | Again, the authors provide a clear (though broad) definition of the types of community-based interventions that are of interest and importantly are clear about what interventions will be excluded and why. I have some concerns about the scale of the task the authors are setting themselves but, as they are only interested in review articles, I think this will be manageable. | R2 |  | No revision is needed. We agree – this is a big task. We have identified over 160 eligible reviews. |  |
| How the intervention might work | While the interventions might be broad, I encourage the authors to think through a framework through which they could have an effect on community violence. This can include the social-ecological model or the WHO Social Determinants of Health Framework: [Social determinants of health (who.int)](https://www.who.int/health-topics/social-determinants-of-health#tab=tab_1). Both have been used to bring together a diverse array of interventions and how they might affect behaviors and health. | R1 |  | We appreciate the feedback, but do not see any value in doing this. The inventions are extremely diverse and even the social-ecological model would be of little value. See the related response to a similar comment below (ED agreeing with this). |  |
| How the intervention might work | The authors do not describe how the intervention(s) might work but justify this exclusion. This study is a review of reviews and could potentially cover a range of different interventions – therefore it is not realistic to expect them to detail the possible causal pathways of each potential intervention in the protocol. However, I did not notice in Appendix 1 (Coding Protocol) any means to systematically collect this information from the individual reviews (perhaps it will not be available in review papers). The authors may want to consider adding a column to their coding protocol to collect this information (where available) and reporting/discussing if appropriate. | R2 |  | We appreciate the feedback, but do not see the point in coding this. Very few systematic reviews and meta-analyses in criminology provide any discussion of a possible causal pathway. Even older Campbell reviews often don’t have this. In our experience, only newer Campbell reviews will address this issue. As a result, addressing this issue is not one of our goals. |  |
| How the intervention might work | I agree with R2, you can at least propose some example theories of change here, while still noting the difficulty in narrowing down to one given the wide range of interventions that will be included in the review. | ED |  | This is beyond the scope of this paper. To accomplish this, we would need to discuss every conceivable theory of change that has been proposed within criminology. This would be a massive effort and is not a goal of this paper. In addition, if we only provide examples, how would we select which to highlight? |  |
| Why it is important to do this review | The authors provide a clear justification for the review which builds on the previous meta review conducted in 2016. Since this time, additional work has been completed which makes the suggested meta review a useful and timely contribution to the field. | R2 |  | No revision is needed. |  |
| Why it is important to do this review | Why do we need this review from a policy and practice perspective? This section should clearly describe the justification for doing the review and should include two main components.   1. A discussion of existing and ongoing primary research, narrative and systematic reviews, and meta-analyses on the topic, to highlight what has been learned from past efforts as well as to point out any inconsistencies, methodological strengths and weaknesses, and evidence “gaps” that still remain. The contribution of the planned review should be emphasised by clearly stating the unresolved questions and controversies that will be addressed. 2. A brief statement on how the review could inform practice or policy decisions. | ED |  | No revisions made for the following reasons:  First, we think that discussing ongoing primary research would be a massive undertaking, given the broad scope of our review. We also think that doing so contradicts the core logic of meta-analysis.  Second, we are unaware of anyone else doing similar work to this.  Third, we are not trying to fill a “gap” or address a controversy. Rather, we are trying to take stock in an organized way what the current body of systematic reviews say about addressing the problem of community violence. Readers will either find this valuable or they won’t. It is not for us to make that decision for them.  Fourth, we believe that the existing paragraph before “Description of the interventions” addresses the two items.  Fifth, we have an entire section on why we think it is important to do this review. |  |

**Objectives Feedback Reviewer Revised? Author comments ED Use Only**

| - | The objectives of the proposed review are clearly stated and will greatly assist policy makers in making decisions about the nature of future services. | R2 |  | No revision is needed. |  |
| --- | --- | --- | --- | --- | --- |
| - | The aims could be clearer – suggest adding “rigorously evaluated through systematic reviews” to aim 1. | ME |  | Thank you for the suggestion. We have made this edit. |  |
| - | The Objectives are framed in a somewhat unresolved way by using ‘For instance”. Are all these questions going to answered in the review, and if so, do the methods allow for these to all be answered?  The implications sentence can be removed, as all Campbell reviews will need to consider implications for policy and practice, as it is a standardised heading for the final review template. | ED |  | Thank you for the feedback. We have deleted the offending sentences. The “for instances” were not about the objects, per se, but examples of what might be learned by addressing them. |  |

**Methods Feedback Reviewer Revised? Author comments ED Use Only**

| Criteria for considering studies for this review | There is a sense of mismatch between the title and background (which focuses on community violence) and the inclusion criteria (which focuses on crime and/or violence). The way the inclusion criteria are framed potentially sets your review up to include almost any review in criminal justice. I will pose specific questions and raise specific issues by each criterion below. | ED |  | No revision is needed. |  |
| --- | --- | --- | --- | --- | --- |
| Criteria for considering studies for this review | Under ‘Types of interventions’, I am unclear how screeners would determine whether an intervention ‘intended to reduce crime and/or violence’. How can something have an *indirect* versus *direct* intention to reduce crime and/or violence? I think I know what you mean here, but the goal is to be transparent and explicit enough that another review team could replicate your review. How can this be operationalised? For example, could it be by the stated aims of the intervention, by the types of outcomes used, by the type of people/practitioners implementing the intervention, and/or by the type of population targeted? Not all reviews and/or studies are as clear as we would like, and so it needs to be clear how the author team is ascertaining whether an intervention intends to reduce crime and/or violence.  I then became confused regarding the exclusion of interpersonal violence. This is because your definition of community violence explicitly includes interpersonal violence. What exactly do you mean by interpersonal violence here? It may be helpful to consider the staged definition of violence used by WHO (i.e., state, collective, interpersonal, self-directed violence). What about school-based violence? What about sexual violence? | ED |  | This was our mistake. We had intended to say, “intimate partner violence” but wrote “interpersonal violence.” We have fixed this. |  |
| Criteria for considering studies for this review | Under “types of interventions,” the authors state that they will exclude interventions focused on interpersonal violence and domestic violence. Additional clarity is needed in what is meant by “interpersonal violence” because all violence that occurs between individuals is interpersonal, including community violence. | R1 |  | See above. We have fixed this. |  |
| Criteria for considering studies for this review | Under ‘Types of participants’, the statement that “Essentially, any individual is eligible so long as the intervention is eligible” is not needed given your specification of any age, gender/sex etc. You can simply state that any participant type is included if that is your intention. Also include sex, as this is often how sociodemographics are reported in studies/reviews (there is not yet clear/consistent use of gender and sex yet). Will you include practitioners as the participants of the interventions?  I did, however, have some follow-up questions on this specific criterion. This is where some definitional tightening will help if the following example is not your intention. Example: Some would argue that parenting or family therapy programs aim to, in the long-term, prevent crime and/or violence. And given there are no limits on populations, that could mean that you would include a body of literature on therapeutic interventions where parents and/or children/adolescents are the participants. I say this because the definition of the outcome has not covered jurisdictional issues like age of criminal responsibility. Is that the intention? If not, then I strongly urge you to consider the implications of such a large scope and consider placing some boundaries around inclusion at participant and outcome levels. | ED |  | Thank you for your feedback. We have simplified this in light of your comments.  We have changed “gender” to “sex.”  Yes, our inclusion criteria would include therapeutic interventions for families. However, the meta-analysis would need to examine crime as an outcome variable. |  |
| Criteria for considering studies for this review | Under “types of outcome measures:”   - - The authors state their primary outcome of interest is “criminal violence.” All violence is illegal and “criminal.” Are the authors referring to their definition of community violence here or something else?   - The authors also signal their attention to include “community disorder” in their measures. An explicit definition of this is needed here and in Appendix 1 (the coding sheet). Based on the information provided, it is likely that the study team may not reliably capture that information without a definition. | R1 |  | We have made several edits based on this feedback.  Criminal violence has been changed to community violence.  Community disorder is simply any measure that a meta-analyst designates as community disorder.  Disorder (broken windows policing) is listed within a list of many intervention options in our coding database, and it is not appropriate to provide a definition, as none of the other options have definitions either. |  |
| Criteria for considering studies for this review | I strongly agree with R1. What exactly do you mean by criminal violence? That could have a different definition depending on the jurisdiction and so I will require you to explicate your outcomes section much more. There is also a lack of linkage back to community violence, which appears to be the focus of your review. This is not mentioned anywhere in your inclusion criteria. Things to consider here are not just the data collection methods (which you’ve covered well), but what exactly is being measured. Specific issues here:   - Do you mean specific violent offences? What about ages of criminal responsibility, where sometimes violent offences are not recorded as a criminal offence? - Is it the act in question, rather than whether the person has been found to have committed or is suspected of a criminal offence (e.g., arrest, calls-for-service versus convictions and sentencing measures). - The reference to ‘general crime’ in multiple places in this paragraph sometimes with and sometimes without reference to exclusion of person (violent) crimes is a touch confusing. Please use one sentence clearly that explicates that you will only include general measures of crime if they include X offences (if this is what you mean). |  |  | Our eligibility criteria are at the level of reviews, not primary studies. As a result, we don’t have the ability to be highly selective or specific regarding which outcomes are included in their overall summaries.  To clarify, we do mean violent offenses.  There is no way for us to consider the age of criminal responsibility. We have not seen a single meta-analysis in criminology discuss this. Generally, individuals below the age of responsibility are not criminally prosecuted, so they wouldn’t be included in official measures of either general or violent crimes.  We aren’t parsing whether it is arrest, conviction, reincarceration, self-report, or some mix. The criteria are explicit on this point.  By general crime, we mean “any crime.” We have edited the text and hope it is now clear. |  |
| Criteria for considering studies for this review | How do you define community disorder? In addition, please specify how you will operationalise the use of secondary outcomes. Will you only include reviews that have criminal violence AND community disorder, or would you include a review with community disorder AND NOT criminal violence? The former creates limits, as it only allows you to draw conclusions on community disorder based on reviews that also use criminal violence, whereas the latter allows you to draw broader conclusions on the community disorder body of reviews. | ED |  | Thank you for the feedback. We have clarified the language. |  |
| Data collection and analysis: Search | The other resources listed under “Searching other resources” (p. 8) is almost exclusively policing and criminal justice resources. I encourage the authors to also include public health and violence prevention resources on this list including the American Journal of Public Health; Journal of Interpersonal Violence; Trauma, Aggression, and Violence; and Journal of the Association of Public Policy Analysis and Management. I also suggest that they find grey literature via the Prevention Institute, Urban Institute, and similar organizations working in public health. | R1 |  | We appreciate the feedback but are not planning on extending our hand search to these additional journals. We think that our systematic search is sufficiently comprehensive to have identified most eligible systematic reviews (we have over 160, not counting multiple publications of the same review or updates of an early review). We do include several grey literature cites, as listed in the protocol. Our list is now inclusive of the Urban Institute and Prevention Institute. |  |
| Data collection and analysis: Search | I did not see how the authors are proposing to find governmental reports, which may not show up in the databases mentioned. More details are needed there. | R1 |  | NCJRS, which was included in our search, is an excellent source of government reports (both those conducted in the US and otherwise). Criminal Justice Abstract also includes government reports, although less completely. |  |
| Data collection and analysis: Search | I would appreciate an IS looking at the search – particularly whether they have a recommended filter for identifying systematic reviews. No reference is given for the filter suggested. | ME |  | We unfortunately do not have access to an IS. However, one of the authors of this review has extensive experience conducting systematic reviews, including systematic searches. We are also unaware of any MECCIR guideline that requires citations to justify the keyword selection. We assume you are referring to the filter restricting the search to meta-analysis and systematic reviews. Note that we are not using the filters that exist with PsychINFO and MedLine for systematic reviews but rather searching the titles, abstracts, and summary fields (i.e., keywords) for relevant terms. If a title, abstract or summary field does not include one of the terms listed in this filter, it is highly unlikely to be eligible. |  |
| Data collection and analysis: Search | In my last action letter, I suggested a list of electronic academic databases that I would like to have seen in your list of sources. Your response correctly highlighted that there is much crossover between search sources. However, it is very difficult to determine that exact crossover. In fact, my colleagues and I have worked with information retrieval specialists to build software to quantify this. While many databases might index the many of the same titles, they often vary on the frequency of when they index records and the dates they cover. Further, their indexing terms are different. For example, Sociological Abstracts in ProQuest (in your search) and International Bibliography of the Social Sciences (suggested, but not in your search), the dates and types of content coverage differ. I ran an exact search in each database (title(violen* AND youth*)) and different records are captured (based on N results and unique records remaining after de-duplication).  If there is indeed substantial crossover as you say, then this would be easily identified and removed during the duplicate removal process. The databases I suggested have good functionality and would not be onerous to search or export from. I am more than happy to be proven wrong if you can quantify that these sources would not identify anything additional or different to the other sources. Without this though, I am requesting that you, at minimum, at the following sources to your search (fewer than previously suggested):   - International Bibliography of the Social Sciences - Social Services Abstracts - Applied Social Science Index and Abstracts | ED |  | We believe that our list of databases is sufficient for our purpose. Note that we are not relying on indexing terms, so that should not be an issue (although we are searching them).  We have selected all relevant database to which we have assess via our library. The MECCIR standard only requires that you search multiple databases, which we have done. |  |
| Data collection and analysis: Search | In my last action letter, I requested additional grey literature sources. R1 has also asked for more comprehensiveness here. While the sources you have listed cover some grey literature and likely some of the content on these websites, I have yet to identify how, when and what NJCRS and CJA capture re: grey literature sources (e.g., does NJCRS capture everything from the Australian Institute of Criminology, which is systematically indexed in CINCH? What about Criminal Justice Abstracts). I am happy to be proven wrong, but I do not think there is a large amount of impost to search a few more sources which definitively contain systematic reviews. The following sources (fewer than previously suggested) need to be included in your next protocol.   - Australian Institute of Criminology ([here](https://www.aic.gov.au/publications), or I am happy to search for you in CINCH) - What Works Crime Reduction Toolkit (this What Works Centre was commissioned to produce systematic reviews in criminal justice, see [here](https://library.college.police.uk/HeritageScripts/Hapi.dll/search2?CookieCheck=45306.3934933565&SearchTerm=WHAT%20WORKS%20CRIME%20REDUCTION%20SYSTEMATIC%20REVIEW%20SERIES&Fields=S&Media=#&Dispfmt=B&SearchPrecision=10&DataSetName=LIVEDATA). They also included reviews to inform their toolkit, which are cited by interventions, see [here](https://www.college.police.uk/research/crime-reduction-toolkit)) - RAND Documents - The Urban Institute   You may also be interested in the list of reviews here: <https://crimesolutions.ojp.gov/rated-practices/screened-out-meta-analyses-practices>  When updating your website search sources, please include all URLs [C36; R34]. | ED |  | Thank you for your feedback. We have added those on this list that we were not already searching.  We have already searched RAND and Urban and had added this in the protocol. Both are also well-indexed by NCJRS. All of the publications referenced in CrimeSolutions.gov are included in NCJRS as indicated in the prior version of the protocol (when you look at an entry for a meta-analysis, it includes the NCJ number as part of the reference citations; this is the NCJRS database ID number).  Criminal Justice Abstracts was already on our list.  We have included URLs. |  |
| Data collection and analysis: Search | I have made some revisions to your search terms via track changes. This is to capture different ways by which ‘systematic reviews’ are captured across disciplines. I also strongly urge you to consider expanding the second clause to capture variants of the violence concept (e.g., what if reviews  In reference to the Methods Editor comment regarding the search filter for identifying reviews. While there has been development and testing of filters for specific databases, mainly in medicine, these have yet to be tested and developed in social sciences. | ED |  | Thank you for your feedback. We have accepted your edits to the search terms.  Our testing of the second set of terms suggests that it reasonably balances sensitivity and specificity. Furthermore, we are unaware of any additional terms that would be useful that would not at the same time return tens of thousands of hits (some that we tried had this issue). |  |
| Data collection and analysis: Search | It is a mandatory standard [C30] to harvest the reference lists of all included studies (in your case, reviews). Please include this step in your next version. | ED |  | We unfortunately do not have the resources to add this to our workload (we are already over budget on this project). Furthermore, the new methods standards that have been vetted by both the methods group and all editors recommend this but do not mandate it. |  |
| Data collection and analysis: Search | In the last action letter, I requested an exact search for one of your search sources and this was indicated as being in Appendix 1, but this does not seem to have been added there. Please add this to the next version of the protocol. | ED |  | Sorry, we thought we’d submitted this. It has now been included as Appendix 1. |  |
| Data collection and analysis: Data extraction | For data extraction, I strongly encourage the authors to do an equity assessment of the evidence. This can help to identify those interventions that might help reduce disparities in community violence, especially due to the fact that there are stark racial/ethnic disparities in violence. | R1 |  | Unfortunately, too few meta-analyses have any information on this to be of any value. We are also not sure how to do this at the meta-review level. Some reviews will provide a moderator analysis comparing majority vs. minority effects. However, this isn’t what equity is about for many interventions. |  |
| Data collection and analysis: Data extraction | Section 3.4 on data extraction is confusing as I think the authors have use “study” to refer to both reviews and to primary studies within reviews. So does “study level characteristics” refer to reviews or included primary studies? | ME |  | Yes, we mean “review” and “results.” We have made these edits. |  |
| Data collection and analysis | The main issues relate to how the review authors intend to identify and account for overlap between studies that are included in different reviews. The authors need to consider and describe this. As you note, overviews of systematic reviews are likely to encounter studies which appear in more than one of the included reviews – which can lead to biases or inaccurate estimates of the amount of evidence available on a particular population, setting, or intervention, or lead to overlapping effect estimates (giving undue weight to a study included in multiple reviews). There may also be differences in the way that study authors have assessed individual studies. This may need to be considered at the study selection, data extraction and synthesis stages – see for example Lunny et al 2021 *Managing overlap of primary study results across systematic reviews: practical considerations for authors of overviews of reviews* for a discussion of the issues, and potential strategies to address these. I’d also encourage them to think about formal assessments of overlap in the reviews. At the least, there is currently insufficient information about how overlap will be considered when synthesising and presenting the findings. | ME |  | We have a full paragraph that addresses this issue, and it is unclear from your comment what was unclear about our proposed method. We are familiar with the method proposed by Lunny et al. and one of the authors of this proposal (DBW) used essentially the same method in his dissertation that synthesized the results from 319 meta-analyses. In that dissertation, a precise index of the overlap was needed given the statistical analyses used. In this overview review, however, we are not performing any synthesis across meta-analyses.  We do not believe that comparing the reference lists across reviews is necessary or feasible for several reasons.   1. We have already identified over 160 eligible reviews. Comparing each with each other would amount to making 12,720 comparisons. Even if many of these pairwise comparisons might logically not be unnecessary (e.g., a hot spots policing review versus a CBT review), a decision must be made for each of these pairs. (DBW: In my dissertation, this task alone took over two months of full-time work.). With an average of 40 studies per review, the number of references that must be compared is staggering. 2. We are not performing any statistical analyses, so a precise number is unnecessary. 3. Not all systematic reviews include the references of the reviewed studies, particularly for large reviews (historically, journals, including APA journals, were unwilling to publish these if they were over 50 references and web-accessible supplemental materials are a relatively new phenomenon). 4. A reasonable and conservative inference regarding overlap can be made in most cases. For example, it is safe to assume that a meta-analysis of CBT reviews has no overlap with a meta-analysis of hot spot policing. Similarly, two meta-analyses of hot spots policing likely have nearly 100 percent overlap, or at least one can conservatively assume that all of the studies from an older and smaller meta-analysis are likely to be included in a new and larger one. The overlap may be less than 100 percent, given different eligibility criteria, but we will assume both are drawing from the same literature.   We have added some additional text that we hope clarifies our approach. |  |
| Data collection and analysis | Additional comments – P. 5 item 6 . 1990 is quite early – reviews published at this date are likely to contain studies of interventions from perhaps 10 or more years previously. Will studies that are 40 or 50 years old be relevant to current understandings? | ME |  | This is a reasonable point. However, we have already done the search and are about halfway through double coding. |  |

**Other comments Feedback Reviewer Revised? Author comments ED Use Only**

| Any other general comments | Appendix 1: What is the “RNR Framework” mentioned under “intervention type?” Please provide a definition of it there. | R1 |  | RNR framework is one option in our database for type of intervention. We have spelled out the acronym to now read “Risk-Need-Responsivity (RNR) framework.” This is a well-known acronym in corrections research. Since the listing of RNR framework is within a list of many intervention options in our coding database, it is not appropriate to provide a definition, as none of the other options have definitions either. This is now in Appendix 2. |  |
| --- | --- | --- | --- | --- | --- |
| Any other general comments | In addition, overviews of reviews are often more complex than anticipated – the focus of the overview authors isn’t always in line with the previous review authors, and so you may find yourself needing to go back to the original included studies for some details – such as greater detail on the population, setting, intervention design etc. As you aim to identify interventions that are effective, you may want to do this if the reviews do not provide sufficient information about what the successful interventions consist of. | ME |  | We unfortunately do not have the capacity to go back to the primary studies included in these reviews. |  |
